# Impulsivity, Lack of Premeditation, and Debts in Online Gambling Disorder

**DOI:** 10.3389/fpsyt.2020.618148

**Published:** 2021-01-20

**Authors:** Isabel López-Torres, Leticia León-Quismondo, Angela Ibáñez

**Affiliations:** ^1^Foundation for Biomedical Research, Hospital Universitario Ramón y Cajal (FIBioHRC), Madrid, Spain; ^2^Department of Psychiatry, Hospital Universitario Ramón y Cajal, Madrid, Spain; ^3^Ramón y Cajal Institute for Health Research (IRYCIS), Madrid, Spain; ^4^Network Centre for Biomedical Research in Mental Health (CIBERSAM), Instituto de Salud Carlos III, Madrid, Spain; ^5^Departamento de Medicina y Especialidades Médicas, Universidad de Alcala, Madrid, Spain

**Keywords:** gambling disorder (GD), online gambling, offline gambling, sports betting, impulsivity (IMP), lack of premeditation, debt, pathological gambling

## Abstract

**Background and Objectives:** Gambling disorder (GD) is a recurrent and persistent problematic gambling behavior that impairs multiple areas of an individual's life. GD can persist through two modes: online or offline. This study aims to compare sociodemographic, clinical, and psychological characteristics between treatment-seeking online and offline gamblers and analyze the effect of the gambling mode (online or offline) on anxiety, depression, impulsivity, and debts.

**Methods:** Seventy-nine treatment-seeking gamblers (96.2% males), who were simultaneously receiving treatment at a specialized Pathological Gambling and Behavioral Addictions Unit, participated in this study. The sample was divided into two subsamples: online (*n* = 29, 100% males) and offline (*n* = 50, 94% males); the characteristics of these two groups were compared and analyzed using Chi-Square test (χ^2^), *t*-Test or Mann–Whitney *U*-test (*p* < 0.05). Multiple linear regression analyses were performed to determine the effects of gambling mode on significant variables (lack of premeditation and debts).

**Results:** The online sample with a mean age of 29.4 years mainly chose to engage in sports betting (45%, *p* < 0.05) and showed a higher lack of premeditation levels (25.8 points, *p* < 0.05) than the offline sample. In addition, the online sample was younger with respect to their onset to gambling (20.2 years, *p* < 0.05) and the beginning of their gambling problems (25 years, *p* < 0.05) compared to the offline sample. Online gambling increased the levels of lack of premeditation by an average of 5.43 points compared to offline gambling (*p* < 0.05). Accumulated debts of the online sample were lower (€11,000) than those of the offline sample (€12,000). However, the interaction between age and gambling mode revealed that online gamblers increased their debt amounts with age at an average increase of €2,726.33 per year compared to offline gamblers (*p* < 0.05). No significant influence of gambling mode was found on GD severity, anxiety, and depression levels.

**Conclusions:** Gambling mode has a significant relationship with lack of premeditation—a component of impulsivity—and accumulation of debts in treatment-seeking people with GD; however, no relationship was found with the rest of the variables analyzed. Future research with larger samples is needed to confirm these findings.

## Introduction

Gambling disorder (GD) has been defined as a recurrent and persistent gambling behavior that deteriorates multiple areas of an individual's life and generates significant emotional distress (1). Such maladaptive gambling behavior can occur in two modes: online (i.e., on the Internet) and offline (2). In Spain, the probability of adults developing gambling-related behaviors is 4.4%, 1% for problem gambling, and 0.9% for GD throughout their lifespan (3), whereas for Spanish adolescents, 8.2% could be considered at-risk gamblers, 5.6% problem gamblers and 1.84% pathological gamblers (4, 5).

Modern technology has led to the unprecedented development and expansion of gambling activities, primarily through online gambling. Recently, there has been an increase in the number of online gamblers in Spain. According to the Directorate General for the Regulation of Gambling (DGOJ), an organization that regulates gambling nationwide in Spain, 83.46% of those who gambled online ranged between 18 and 45 years in 2018 (6). Structural characteristics (ease of betting, immediacy of the prize, and high probability of winning) and immediate infrastructure and environment (privacy, comfort, availability, and accessibility) make online gambling more addictive than offline (5). Problem gambling is more common among online gamblers, especially among vulnerable individuals (7, 8). Gambling advertising and promotion contribute to increased demand for and indulgence in gambling (9). Most gambling advertisements on television are concerned with online gambling (10), which has a significant impact on the probability of developing GD (8). Currently, online gambling, mainly sports betting, acts as the main cause of GD among treatment-seeking patients (11). In Spain, online sports betting has grown rapidly in recent years, contributing largely to the gambling industry's profits. Since 2012, more than half of the online gamblers have indulged in sports betting (52.2%) (12); it has also become a frequent gambling activity among the younger population (13).

GD has often been associated with impulsive behavior and it is considered a risk factor in its etiology (14). Impulsive behaviors in childhood have predicted problem gambling in adulthood (15). A systematic meta-review conducted by Lee et al. (16), showed that impulsivity is a fundamental process underlying addictive behaviors, with and without substance, especially in alcohol abuse and GD. In line with these findings, high impulsivity has been pronounced in people with GD as opposed to healthy controls (17). There are many models that have tried to explain impulsivity and its complex nature (14, 18). The UPPS-P Impulsive Behavior Scale, developed by Whiteside and Lynam (19) and modified by Cyders et al. (20) measures five personality dimensions that contribute to impulsive behavior.

In addition to impulsive behavior, GD is often related to higher stress, anxiety, depression (21, 22), GD severity (23), and debt levels (10). In some instances, the psychological distress may even be mediated by financial debt (24). In addition to the personal economic cost, it has a high sanitary cost related to treatments. A German study estimated an added increase of €27.24 million per year in their health sector, fundamentally caused by increasing gambling problems among online gamblers (25).

To the best of our knowledge, there are few studies that have focused on sociodemographic, clinical, and psychopathological differences between samples of online and offline pathological gamblers. In recent years, there has been a growing interest in the study of GD, especially since the expansion of the online gambling industry and increase in the number of online gamblers.

Previous studies have shown significant differences in sociodemographic variables, such as age and education level, with respect to the gambling mode (26, 27). Studies conducted with samples of pathological gamblers have also found significant differences in GD severity, psychological distress, and personality traits, comparing strategic gambling which emphasize the importance of individual skills (poker, craps, or sports betting) and non-strategic gambling which emphasize chance as playing a bigger part (lotteries, bingo, or slots-machine) (28) or samples of online and offline sports betting gamblers with another sample of general offline gamblers (29).

With respect to impulsivity variables, strategic gamblers have shown a higher lack of perseverance levels than non-strategic gamblers (30). However, they showed similar scores on all impulsivity variables when online and offline gambling samples were compared (30). In addition, high negative urgency levels (31), online gambling, and high levels of debts were identified as predictors of dropout in a cohort of pathological gamblers seeking treatment (32).

Given the context of and increasing number people indulging in online gambling and its addictive component, the objective of the present study was to explore and compare sociodemographic, clinical, and psychopathological variables between two treatment-seeking samples of persons with GD and estimate the effect of gambling mode on GD severity, anxiety, depression, impulsivity, and accumulated debts. We hypothesized higher GD severity, impulsivity, accumulated debts, anxiety, and depression levels in online gamblers compared to offline gamblers. Considering that online gambling is more harmful than offline gambling, we expected to be able to estimate the effect of gambling mode on the variables under study. Additionally, given that most online samples indulged in sports betting, we wanted to explore its effect on online gamblers.

## Materials and Methods

### Participants

The participants were pathological gamblers, who were treated together in the Pathological Gambling and Behavioral Addictions Unit of the Ramon y Cajal University Hospital (Madrid, Spain), between January 2019 and March 2020. The inclusion criteria were a diagnosis of GD according to DSM-5 criteria and over 18 years of age. Comorbidity with intellectual disability; history of substance abuse/dependence; diagnosis of schizophrenia, or other psychotic disorders, major depression, or bipolar disorder, as well as severe organic and/or neurological pathology including history of traumatic brain injury, or epilepsy were set as exclusion criteria. The initial sample consisted of 102 patients, 23 of which were excluded because of comorbidities; thus, the final sample comprised of 79 patients. The total sample was divided into online (inclusion criteria: gambling predominantly or exclusively online) and offline (inclusion criteria: gambling predominantly or exclusively offline) gamblers. Finally in our sample all the players included in the offline sample played exclusively offline, none of them had any problems with the online game.

### Measures

#### Diagnostic Questionnaire for Pathological Gambling According to the DSM-5 Criteria

This is a self-report questionnaire developed to identify the presence of GD according to DSM-5 (1). It has 19-item based on the DSM criteria. The total scores range from 0 to 10. The cutoff point was 4 or more (33). It is a reliable, valid, and accurate instrument for GD diagnosis (Cronbach's alpha for our sample, α= 0.85).

#### South Oaks Gambling Screen (SOGS)

It is a screening instrument that is used in many studies as a measure of severity of gambling activity. It has 20-item, which a total scores range from 0 to 20. The cutoff point was 5 or more, indicating a probable pathological gambler (34, 35). This study used the Spanish version of the scale, which showed high internal consistency in our sample (Cronbach's alpha, α= 0.85).

#### Yale-Brown Obsessive Compulsive Scale Adapted for Pathological Gambling (PG-YBOCS)

PG-YBOCS measures the severity and change in GD symptoms over a recent time interval (usually within the past 1 or 2 weeks). This is a 10-item scale, divided into two subscales (gambling thoughts or urges and gambling related behavior) and an overall symptom severity score. The total scores range from 0 to 40 (36). It is a reliable and valid instrument for GD severity, showing high internal consistency (Cronbach's alpha, α= 0.85).

#### State-Trait Anxiety Inventory (STAI)

This questionnaire evaluates anxiety as a state (momentary, transitory) and as a trait (more stable condition). It comprises of 40 items divided into two subscales: trait and state, with Likert-type responses from 0 to 3. The total score for each subscale ranges from 0 to 60. There is no cut-off point (37). This study used the Spanish version of the scale, which showed high internal consistency in our sample (trait anxiety Cronbach's alpha, α= 0.85, and state anxiety Cronbach's alpha, α= 0.85).

#### Beck Depression Inventory-Second Edition (BDI-II)

This questionnaire measures the severity of depression in adults and adolescents aged 13 and older. It comprises of 21 items. The total score ranges from 0 to 63 points. The following cut-off points were established: 0–13, minimal depression; 14–19, mild depression; 20–28, moderate depression; and 29–63, severe depression (38, 39). This study used the Spanish version of the scale, which showed high internal consistency in our sample (Cronbach's alpha, α= 0.85).

#### The UPPS-P Impulsive Behavior Scale

This scale measures five personality dimensions that contribute to impulsive behavior: negative urgency (tendency to lose control under negative emotions), positive urgency (tendency to lose control under positive emotions), sensation seeking (predisposition to try new and stimulating activities), lack of premeditation (tendency to make decisions without considering their consequences), and lack of perseverance (inability to maintain the level of effort needed during a demanding task). It has 59 items, which are scored using a Likert-type scale (19, 40). This study used the Spanish version of the scale, which showed high internal consistency in our sample (Cronbach's alpha negative urgency, α= 0.86; positive urgency, α= 0.87; sensation-seeking, α= 0.90; lack of premeditation, α= 0.88; lack of perseverance, α= 0.86).

#### Other Variables

Data about sociodemographic variables such as age, gender, marital status, educational level, and employment were collected. Furthermore, gambling mode and type, onset of gambling activity, onset of GD, GD progression, and accumulated debts were registered using a standardized *ad hoc* questionnaire. All variables were systematically collected from the participants.

### Procedure

This study was approved by the Ethics Committee of the Ramon y Cajal University Hospital and was in accordance with the principles of the Declaration of Helsinki. All patients provided written informed consent for their participation in the study. There was no monetary compensation for their participation.

All the assessment procedures were conducted in a single session where patients completed the self-report questionnaires (for determining GD severity, impulsivity, anxiety, and depression levels), participated in a structured face-to-face clinical interview, and answered a standardized *ad hoc* questionnaire (for reporting sociodemographic and clinical variables).

### Statistical Analyses

The total sample was divided into two groups for the initial analysis: online gambling sample (*n* = 29) and offline gambling sample (*n* = 50). Sociodemographic, clinical, and gambling characteristics (age, sex, marital status, educational level, employment, family gambling history, gambling activity, debts, and impulsivity levels) were explored. Qualitative variables were assessed by absolute and relative frequency and differences between samples were assessed using the chi-square test (χ^2^). Continuous variables were assessed using mean and standard deviation (SD) in variables with normal distribution, or median and interquartile range (IQR) in non-normal distribution variables; differences between samples were explored using a *t*-test or Mann–Whitney *U*-test, respectively. All contrasts were bilateral, with a significance level of *p* < 0.05.

Multiple linear regression analyses were performed to determine the effects of gambling mode and gambling type on the variables that showed significant differences between the groups (lack of premeditation and accumulated debts). Due to significant differences between participants' age in the online and offline groups, all models were adjusted for age. The method to introduce variables in models was stepwise and was completed from the beginning. As both age and gambling mode had significant effects on accumulated debt, the interaction effect was studied to assess the effect of gambling mode on different ages. Statistical analyses were carried out using SPSS (Version 26) and STATA (Version 16.1 for Windows).

## Results

### Sample Characteristics

Table 1 includes a description of sociodemographic, gambling type, debts and impulsivity variables, and differences found between samples. The online sample comprised of only males, while the offline sample was predominantly males (94%). The demographics showed that online sample had higher levels of unmarried (52%), educated (41%, secondary; 31%, university) and employed (62%) individuals than the offline gambling sample; however, the offline sample had a higher level of family gambling history (22%), than the online sample. The primary significant difference was seen in the age (*p* < 0.05) between the online (M = 29.4 years, SD = 7.6) and offline (M = 46.8 years, SD = 15.8) sample. In addition, the results reported a significant difference (*p* < 0.05) in the gambling type between the online (45%, sports betting; 34% sports betting in combination with other gambling types) and offline (54% slot machines, 12% other combinations of offline gambling activity) gambling samples. Lastly, significant differences were detected in one of the impulsivity components, namely, lack of premeditation levels as per the scores of the UPPS-P subscales (online = 25.8, offline = 22, *p* < 0.05). No other significant differences were found in any variables.

**Table 1:** Online and offline gambler characteristics.

		**Online gamblers (*n* = 29)**	**Offline gamblers (*n* = 50)**	***P*-value**
Age (years), mean (SD)		29.4 (7.6)	46.8 (15.8)	0.000^*^
Gender				
	Men	29 (100%)	47 (94%)	^***^
	Women	0 (0%)	3 (6%)	
Marital status				
	Single	15 (52%)	14 (28%)	0.072
	Married-couple	9 (31%)	28 (56%)	
	Divorced-separated	3 (10%)	5 (10%)	
	Lost values	2 (7%)	3 (6%)	
Education level				
	Primary	6 (21%)	16 (42%)	0.225
	Secondary	12 (41%)	14 (37%)	
	University	9 (31%)	8 (21%)	
	Lost values	2 (7%)	0 (0%)	
Employment				
	Employed	18 (62%)	28 (56%)	0.837
	Unemployed	10 (35%)	14 (28%)	
	Lost values	1 (3%)	8 (16%)	
Family gambling history				
	No family gambling history	25 (86%)	39 (78%)	0.613
	First degree family	2 (7%)	6 (12%)	
	Second degree family	2 (7%)	5 (10%)	
Gambling type				
	Slot machine	0 (0%)	27 (54%)	0.000^*^
	Sport betting	13 (45%)	7 (14%)	
	Roulette	4 (14%)	6 (12%)	
	Other (e.g., bingo, lottery, poker)	2 (7%)	4 (8%)	
	Two or more types	10 (34%)	6 (12%)	
Debts				
	Yes	24 (83%)	33 (66%)	0.109
	No	5 (17%)	17 (34%)	
Accumulated debts (€) median (IQR)^**^		11000 (5000; 35000)	12000 (3500; 21000)	0.059
UPPS-P impulsive behavior scale				
	Negative urgency, mean (SD)	30.2 (6.7)	33.3 (9.0)	0.236
	Positive urgency, mean (SD)	27.5 (7.8)	34.4 (13.5)	0.062
	Sensation-seeking, mean (SD)	31.8 (8.7)	27.2 (9.7)	0.129
	Lack of premeditation, mean (SD)	25.8 (4.7)	22.0 (6.3)	0.040^*^
	Lack of perseverance, mean (SD)	22.5 (3.8)	20.8 (6.0)	0.325

*SD, standard deviation; IQR, interquartile range; UPPS-P, Impulsive Behavior Scale*.

* *p < 0.05*;

** *Median and range have been calculated only in patients with debts*.

*** *The distribution by gender is shown for the purposes of sample description. Statistical analysis cannot be performed because of the small sample size in women, who are only represented in the offline sample*.

### Activity Onset, GD Onset, GD Progression and Clinical Profile

Table 2 contains a description of the onset of gambling activity, onset of GD, GD progression, and other clinical variables (GD severity, anxiety, and depression levels). Significant differences were found between samples in terms of gambling activity onset (*p* < 0.05), where online gamblers were on average 7.43 years younger than offline gamblers at the onset of gambling activity. At the onset of GD (*p* < 0.05), online gamblers were on average 8.89 years younger compared to the offline gamblers.

**Table 2:** Activity onset, GD onset, GD progression, and clinical profile.

	**Online gamblers (*n* = 29)**	**Offline gamblers (*n* = 50)**	**Coefficient (CI 95%)**	***P*-value**
Gambling activity onset (age, years), mean (SD)	20.2 (4.2)	27.6 (13.7)	7.43 (1.99; 12.86)	0.008^*^
GD onset (age, years), mean (SD)	25.0 (7.1)	33.6 (14.1)	8.89 (2.96; 14.83)	0.004^*^
GD progression (age, years), mean (SD)	4.8 (6.5)	6.0 (10.1)	1.16 (−3.28; 5.61)	0.604
DSM-5 total criteria	7.3 (2.0)	7.4 (2.0)	0.02 (−0.95; 1.00)	0.959
SOGS total	11.6 (3.1)	11.06 (2.7)	−0.56 (−1.95; 0.82)	0.417
PG-YBOCS total	17.8 (10.0)	16.8 (9.4)	−1.06 (−5.77; 3.64)	0.653
BDI-II	20.4 (17.7)	18.9 (13.7)	−1.48 (−11.06; 8.10)	0.756
STAI-state	26.6 (18.0)	24.2 (14.1)	−2.37 (−12.28; 7.54)	0.632
STAI-trait	28.2 (15.8)	26.0 (13.2)	−2.18 (−11.29; 6.93)	0.631

*SD, standard deviation; CI, confidence interval; GD, Gambling disorder; SOGS, South Oaks Gambling Screen; PG-YBOCS, Pathological Gambling Yale-Brown Obsessive Compulsive Scale; BDI-II, Beck Depression Inventory-Second Edition; STAI, State-Trait Anxiety Inventory*.

* *p < 0.05*.

No statistically significant differences between the groups were found for GD progression (*p* = 0.604), DSM-5 total criteria (*p* = 0.959), SOGS total (*p* = 0.417), PG-YBOCS total (*p* = 0.653), BDI-II (*p* = 0.756), STAI-state (*p* = 0.632), and STAI-trait (*p* = 0.631).

### Effect Estimation Models

Estimation results of linear regression models are showed in Table 3.

**Table 3:** Estimation results of linear regression models.

	**Coefficient**	***P*-value**	**95% CI**
**Model 1: Lack of premeditation (UPPS-P)**			
Gambling mode (online)	5.43	0.021^*^	−10.00; −0.86
Age (years)	0.089	0.250	−0.07; 0.24
**Model 2: Lack of premeditation (UPPS-P)**			
Gambling mode (online)	4.84	0.039^*^	0.27; 9.41
Gambling type (sports betting)	2.96	0.136	−0.98; 6.91
Age (years)	0.07	0.329	−0.08; 0.23
**Model 3: Accumulated Debts (€)**			
Gambling mode (online)	−63345.59	0.036^*^	−122313.5; −4377.68
Gambling type (sports betting)	5711.50	0.530	−12332.2; 23755.92
Age (years)	240.78	0.416	−346.77; 828.32
Online gambling, age (years)	2726.33	0.003^*^	984.27; 4468.39

*CI, confidence interval; UPPS-P, Impulsive Behavior Scale*.

* *p < 0.05*.

Model 1 contains the effect of gambling mode on the lack of premeditation. Online gambling, as the main effect on lack of premeditation, was an explanatory variable (*p* < 0.05). Online gambling increased the levels of lack of premeditation by an average of 5.43 points compared to offline gambling.

Model 2 shows the effect of lack of premeditation in online gambling, controlling for sports betting and age. Only online gambling had a significant effect on lack of premeditation which increased on average 4.48 points compared to offline gambling (*p* < 0.05).

Model 3 displays the effect of online gambling and sports betting on accumulated debts (only in patients with debts), and the interaction effect between gambling mode and age. Gambling mode had a significant main effect on accumulated debts (*p* < 0.05). The interaction effect between age and gambling mode showed that online gamblers' amount of debt increases with age, with an average increase of €2,726.33 per year compared to offline gamblers.

## Discussion

This study examined and compared sociodemographic, clinical, and psychopathological variables between two patient samples (online and offline gamblers) seeking treatment for GD. The main hypothesis was that online and offline gambler profiles would be different and that online gamblers would present higher GD severity, impulsivity, anxiety and depression levels, and accumulated debts than offline gamblers.

### Sociodemographic Variables

The results of this study showed that there are significant age differences between the online sample, (M = 29.4 years) compared to the offline sample (M = 46.8 years). This can be attributed to the legalization of online gambling, which has led to an increase in the number of pathological gamblers, especially among young adults. Of the online gamblers in Spain, 34.41% are between 26 and 35 years (6). In this study, the onset of gambling activity in the online sample was at a younger age (about 20 years) than in the offline sample (about 27 years). Similarly, the age at onset of GD in online gambling was lower than that in the offline sample. Although online gamblers are not a homogeneous group, since different types of behavior can be considered in the online gambling sphere (41), there is evidence that increased participation in online gambling increases the likelihood of developing GD (8).

Relative to the type of game, the differences between the samples are also notable. Sports betting is the most practiced type of gambling among online sample. Previous literature provides evidence of different phenotypes of online sports betting gamblers (42). In contrast, among offline gamblers, slot machines are the most common gambling activity. Until the advent of online gambling, slot machines were largely responsible for GD behaviors due to their high addictive levels (43).

The finding that online gamblers were younger than offline gamblers could be partly explained by the fact that the advertising and promotion of online gambling, mostly related to sports betting, targets young adults. There are studies that link the type of gambling to the emergence of gambling advertising. For example, in a sample of Swedish online gamblers, casino games were the most advertised and practiced compared to other types of gambling (10). Sports betting advertisements seek to normalize betting activity (43), by highlighting positive aspects (44). Young adults are beginning to bet on well-known soccer, tennis, or basketball competitions (13). Based on the small number of women in our sample, we were unable to assess gender differences. Previous literature has showed that women are less represented than men in clinical samples (45).

### GD Severity

The results reported no statistically significant differences GD severity between online and offline samples. In line with our results, a study also compared sociodemographic and clinical characteristics of treatment-seeking GD patients; no differences were found in DSM-5 and SOGS severity levels between offline gamblers, sports online and non-sports online gambling groups (29). In another study, which compared strategic and non-strategic treatment-seeking GD, no differences between groups were found either. However, age and age of GD onset were found to be predictive of the severity of the disorder in another (28).

### Impulsivity (Lack of Premeditation)

According to the UPPS-P model, lack of premeditation is the tendency to make decisions without considering their consequences (19). It is known that in people with GD, lack of premeditation is related to unfavorable decision-making (46), such as an inability to identify the possible negative financial problems due to gambling (18). This could be a potential explanation for the lower premeditation scores obtained in online gamblers than offline gamblers in the current sample. Similar results were obtained in another study, where a higher lack of premeditation was found among strategic gamblers, such as sports betting gamblers, compared to non-strategic gamblers (30). This is because sports betting may be considered less harmful or problematic by gamblers than other gambling types. A survey of Canadian teenagers reported that they understood betting (sports betting) and gambling differently, where betting was not considered as gambling (47). Lack of premeditation has also been associated with drinking behavior in daily life (48), and it is considered a risk predictor of problem alcohol use (49). Furthermore, it could increase the likelihood of being a smoker as an adult (50). Nevertheless, according to the results of this study, online gambling would significantly increase the lack of premeditation level than offline gambling. Therefore, the structural characteristics and environmental conditions in which the online gambling takes place (5) could encourage online gamblers to take less premeditated actions, regardless of the gambling type.

### Accumulated Debts

In this study, the accumulated debts between the samples were also compared. Although the difference was not statistically significant, the findings revealed that debts are more common among online than offline gamblers. However, the average amount of debt accumulated in offline gamblers is higher than that in online gamblers. These findings are inconsistent with a previous study carried out in a treatment-seeking gamblers, which found that the highest mean debt for online gamblers was $20,000, compared to $500 for offline gamblers (51). Some online gamblers are in debt or over-indebted (10). The current results are consistent variables such as age are considered, for instance, since online gamblers are younger than offline gamblers, they have lesser time to accumulate more debt. In addition, it could be explained by the fact that among online gamblers, the percentage of unemployment is higher than in the offline sample, thus, one possibility is that online gamblers have less money to spend in gambling. Lastly, the annual debt increase of €2,726.33 of online gamblers as their age increases may indicate greater involvement and expenditure in gambling.

### Anxiety and Depression

Several studies have reported high levels of anxiety and depression in GD (24, 52). Depression severity has even been predicted by gamblers involved in multiple online activities (53). The results are in line with these findings with high scores on the STAI and BDI-II. However, no differences in anxiety and depression levels were found between online and offline samples, which counters the initial hypothesis that online gamblers would have higher levels for both. STAI and BDI-II scores were high in both samples, which indicates high psychological stress related to GD behaviors.

### Strengths and Limitations

The results of this study are preliminary, and its major strengths are the use of standardized instruments and the study of patients in a treatment program. However, this study also has some limitations that should be considered. The main limitation is the sample size, which is a limiting factor in the power of the statistical analyses and effect size. Due to the limitations of the sample size, the analyses were focused on the significant results obtained in variables of clinical interest by comparing the samples (anxiety, depression, lack of premeditation, and debt). Therefore, no correction methods were employed for the multiple comparisons to avoid losing the statistical power and increasing the type II error. Another limitation of this study is the underrepresentation of women; thus, gender differences could not be explored. Exploring impulsivity and lack of premeditation through a single measure is also a limitation; therefore, future research should include a more comprehensive assessment, which also includes a non-gambling control sample. In addition, this study was not specifically designed to explore the interaction between the mode and type of gambling; thus, its analysis can only be considered preliminary.

## Conclusion and Implications

In conclusion, the results obtained in our study suggest that the gambling mode (online or offline) could be related to impulsivity and accumulated debts in treatment-seeking people with GD. Thus, online gambling was associated with higher levels of lack of premeditation and lower accumulated debts. However, the amount of debt of online gamblers increases with age increases as compared to offline gamblers.

No influence of gambling mode was found on GD severity, anxiety and depression levels, or other components of impulsivity, such as negative and positive urgency, sensation-seeking, or lack of perseverance.

Future researchers should increase the sample size, including subsamples of online, offline, and mixed gamblers (gambling both online and offline), who might show differentiated clinical and psychological characteristics, and with adequate gender representation. The design of future studies should also include a greater representation of the different types of gambling within each gambling mode (online vs. offline) to specifically explore the interaction between these two variables in GD. Finally, future research might be able to help design psychotherapeutic treatment programs that are more adjusted to patients' needs. It would be interesting to devise treatment programs that place special emphasis on the lack of premeditation to achieve greater control over one's own behavior as well as a reduction in the harmful effects caused by gambling, such as the amount of accumulated debts among people with GD.

## Data Availability Statement

The datasets generated for this article are not readily available because they belong to the hospital database and are not available for public access in order to protect patient confidentiality and in accordance with the informed consent signed by the patients. Requests to access the datasets should be directed to Angela Ibáñez, angela.ibanez@uah.es.

## Ethics Statement

The studies involving human participants were reviewed and approved by Ethics Committee of the Ramon and Cajal University Hospital. The patients/participants provided their written informed consent to participate in this study.

## Author Contributions

AI outlined the conceptualization. IL-T, LL-Q, and AI were responsible for organizing the fieldwork and took part in the data analyses and contributed to the interpretation of the results. IL-T and LL-Q were responsible for the preparation and checks of the data. IL-T drafted the initial version of the manuscript. All authors revised the manuscript for important intellectual content and approved the final manuscript.

## Conflict of Interest

The authors declare that the research was conducted in the absence of any commercial or financial relationships that could be construed as a potential conflict of interest.

## Abbreviations

GD: Gambling Disorder
UPPS-P: Impulsive Behavior Scale
SOGS: South Oaks Gambling Screen
PG-YBOCS: Pathological Gambling Yale-Brown Obsessive Compulsive Scale
BDI-II: Beck Depression Inventory-Second Edition
STAI: State-Trait Anxiety Inventory.
